# Glycated Hemoglobin Independently Predicts Stroke Recurrence within One Year after Acute First-Ever Non-Cardioembolic Strokes Onset in A Chinese Cohort Study

**DOI:** 10.1371/journal.pone.0080690

**Published:** 2013-11-13

**Authors:** Shuolin Wu, Yuzhi Shi, Chunxue Wang, Qian Jia, Ning Zhang, Xingquan Zhao, Gaifen Liu, Yilong Wang, Liping Liu, Yongjun Wang, On Behalf of the Investigators for the Survey on Abnormal Glucose Regulation in Patients With Acute Stroke Across China (ACROSS-China)

**Affiliations:** Department of Neurology, Beijing Tiantan Hospital, Capital Medical University, Beijing, China; College of Pharmacy, University of Florida, United States of America

## Abstract

**Objective:**

Hyperglycemia is related to stroke. Glycated hemoglobin (HbA1c) can reflect pre-stroke glycaemia status. However, the information on the direct association between HbA1c and recurrence after non-cardioembolic acute ischemic strokes is rare and there is no consistent conclusion.

**Methods:**

The ACROSS-China database comprised of 2186 consecutive first-ever acute ischemic stroke patients with baseline HbA1c values. After excluding patients who died from non-stroke recurrence and patients lost to follow up, 1817 and 1540 were eligible for 3-month and 1-year analyses, respectively. Multivariate Cox regression was performed to evaluate the associations between HbA1c and 3-month and 1-year stroke recurrence.

**Results:**

The HbA1c values at admission were divided into 4 levels by quartiles: Q1 (<5.5%); Q2 (5.5 to <6.1%); Q3 (6.1% to <7.2%); and Q4 (≥7.2%). The cumulative recurrence rates were 8.3% and 11.0% for 3 months and 1 year, respectively. In multivariate analyses, when compared with Q1, the adjusted hazard ratios (AHRs) were 2.83 (95% confidence interval (CI) 1.28-6.26) in Q3 and 3.71(95% CI 1.68-8.21) in Q4 for 3-month stroke recurrence; 3.30 (95% CI 1.31-8.34) in Q3 and 3.35 (95% CI 1.36-8.21) in Q4 for 1-year stroke recurrence. Adding fasting plasma glucose in the multivariate analyses did not modify the association: AHRs were 2.75 (95% CI 1.24-6.11) in Q3 and 3.67 (95% CI 1.59-8.53) in Q4 for 3-month analysis; AHRs were 3.08 (95% CI 1.10-8.64) in Q3 and 3.31(95% CI 1.35-8.14) in Q4 for 1-year analysis.

**Conclusions:**

A higher “normal” HbA1c level reflecting pre-stroke glycaemia status independently predicts stroke recurrence within one year after non-cardioembolic acute ischemic stroke onset. HbA1c is recommended as a routine test in acute ischemic stroke patients.

## Introduction

Stroke has surpassed heart disease and become the leading cause of mortality and adult disability in China. The cumulative rate of stroke is 11.2% [1], and the most recent data show that the cumulative acute ischemic stroke (AIS) recurrence rate within 1 year is 17.7% [2] in China, which is apparently higher than that in the Western countries [3]. Hyperglycemia or diabetes mellitus is a known risk factor for stroke recurrence [4,5]. Prediabetes has also widely been considered as a risk predictor for initial stroke [6,7] and impaired fasting glucose is associated with recurrent cardiovascular disease (CVD) [8]. An HbA1c level of ≥6.5% is one of the criteria for diagnosing diabetes mellitus [9], and a range of HbA1c from 5.7% to ≤6.4% was also recommended as the diagnostic criterion for prediabetes by American Diabetes Association in 2012 [10]. Moreover, the baseline HbA1c value at admission to hospital presents the mean plasma glucose level of the 2-3 months preceding acute stroke onset, which reflects pre-stroke glycaemia status (PSGS) [11]. Although HbA1c has been identified to directly associate with CVD incidence [12], the investigation on the relation between the PSGS (measured as HbA1c) and stroke recurrence is rare [13]. Whether the HbA1c level of lower than the HbA1c cutoff point for diabetes diagnosis (6.5%) is independently associated with stroke recurrence still remains unclear. The present study aimed to determine such an association among patients with first-ever non-cardioembolic acute ischemic strokes (AIS) within 1 year after stroke onset. 

## Materials and Methods

### Ethics Statement

The Ethics Committees of Beijing Tiantan Hospital at all participating centers approved the procedures. Written informed consent was obtained from all patients or from the designated family member when the patient was unable to complete it.

### Introduction for ACROSS-China and patient selection

The Abnormal gluCose Regulation in patients with acute strOke acroSS China (ACROSS-China) was a nationwide, multicenter, prospective cohort study that was conducted from August 2008 to October 2009. Patients who did not have a medical history of stroke were recruited consecutively. The inclusion criteria were: acute occurrence within 14 days of neurological deficit with focal or overall involvement of nervous system, including ischemic stroke, intracerebral hemorrhage, and subarachnoid hemorrhage (SAH). The exclusion criteria were: nonvascular causes (primary and metastatic neoplasms, postseizure paralysis, head trauma, and others) that lead to brain function deficit [14]. 

**Figure 1:**
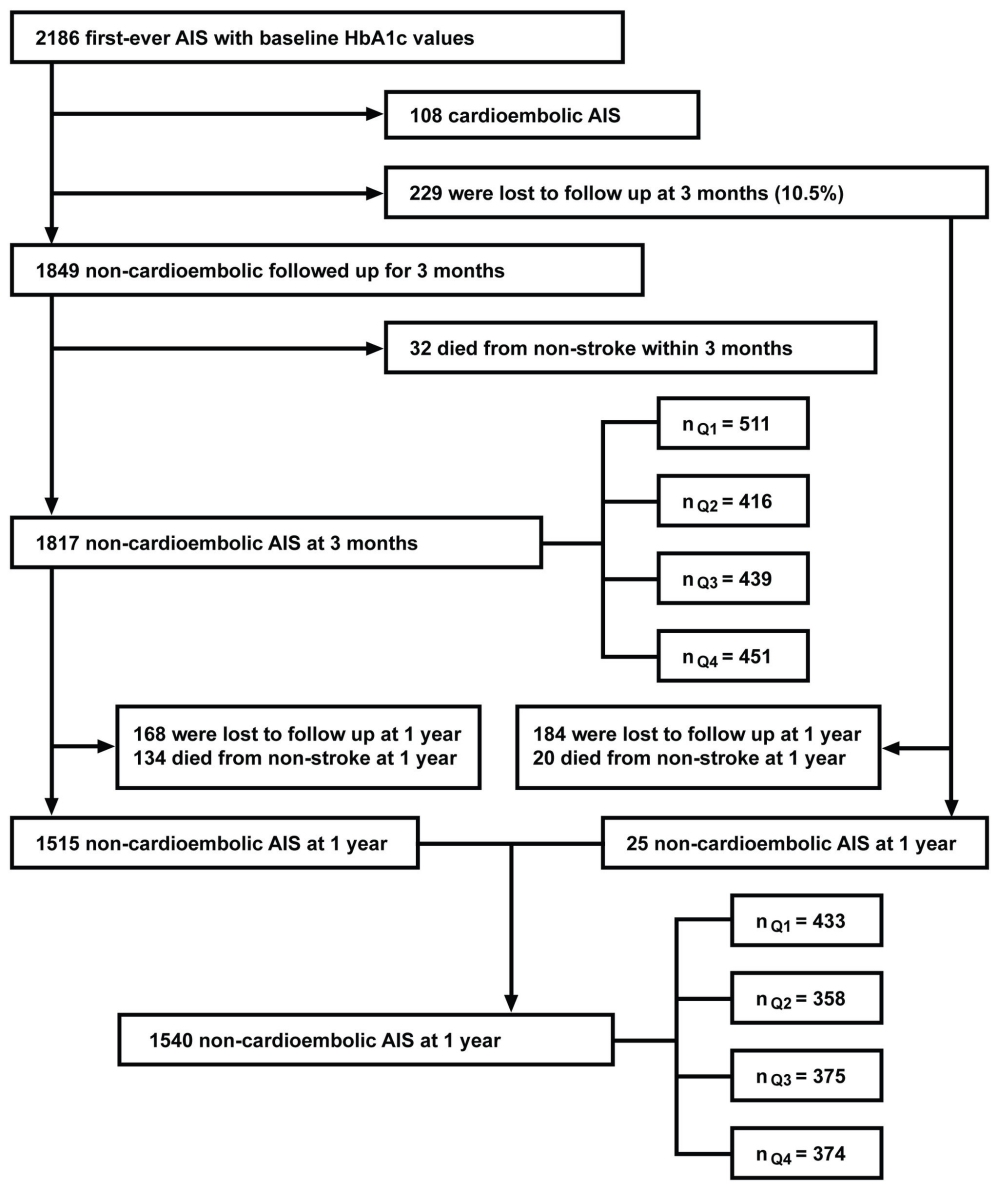
Flow chart of patient selection. AIS indicates acute ischemic stroke; Q1, HbA1c level of <5.5%; Q2, HbA1c level of 5.5 to <6.1%; Q3, HbA1c level of 6.1 to <7.2%; Q4, HbA1c level of ≥7.2%.

The patient selection procedure in the present study was as follows: of all the ischemic stroke patients, those with HbA1c values were included (n=2186); among those, patients with cardioembolic ischemic stroke (n=108), patients who died from non-stroke (n=32 at 3-month follow-up, and n=154 afterward to 1-year follow-up) were excluded, and patients lost to follow up were excluded (n=229 at 3-month follow-up and n=352 at 1-year follow-up). Thus, 1817 patients were available for 3-month analysis and 1540 patients were available for 1 year analysis (Figure 1).

### Demographic and clinical data

All subjects were consecutively enrolled within 14 days after initial stroke onset. Patients' demographic and clinical data were obtained within 24 hours after admission. Clinical data included tobacco use, alcohol intake (moderate or severe drinking, ≥2 standard alcoholic beverages consumed per day), medical history, body mass index (BMI), waist circumference, systolic and diastolic blood pressure, blood biochemical data, and stroke subtype according to the criteria described in the Oxfordshire Community Stroke Project (OCSP) [15] and the Trial of ORG 10172 in Acute Stroke Treatment (TOAST) [16]. OCSP looks at partial anterior circulation infarct, total anterior circulation infarct, lacunar infarction, and posterior circulation infarct. TOAST looks at large vessel infarction, small vessel infarction, cardioembolic, and stroke of other determined or undetermined causes. Classification of stroke subtypes was dependent on clinical history, reliable clinical information, and radiological (computer tomography/magnetic resonance imaging scan) findings. Tobacco use was categorized as ‘current’, ‘previous’ or ‘never’ smoking. ‘Current smoking’ was defined as an individual who smoked at the time of stroke. ‘Previous smoking’ was defined as an individual who had quit smoking within 1 year. ‘Never smoking’ was defined as an individual who never smoked. Blood biochemical data consisted of HbA1c, fasting plasma glucose (FPG), fasting insulin, high density lipoprotein, low density lipoprotein, creatinine, uric acid, and homocysteine. The fasting insulin and FPG values were used to generate the insulin-resistance index (the correctly solved computer model for homeostasis model assessment of insulin resistance, HOMA2-IR) [17]. Data on medications including anti-thrombotic agents (aspirin or clopidogrel), anti-hypertension agents (calcium channel blocker, angiotensin-converting enzyme inhibitors, diuretic, angiotension receptor blocker, or beta receptor blocker), and lipid-lowering agents (statins) were recorded during hospitalization and at 3-month and 1-year intervals. If an individual received the medication during hospitalization, ‘yes’ was recorded. Medication adherence was calculated as the cumulative duration of taking drugs (antithrombotic, antihypertensive, or lipid-lowering therapy) divided by the length of the follow-up period. If the patient experienced stroke recurrence, adherence would be calculated as the cumulative duration of taking drugs divided by the length of the follow-up period before the recurrence event. Medication adherence of ≥ 75% was defined as ‘high’ and < 75% was defined as ‘low’. Patients who did not take any medication during follow-up period were defined as ‘untreated’. A detailed description on medication adherence is provided in Text S1. HbA1c values were measured at admission using ‘high performance liquid chromatographic analysis’ (HPLC) by a Bio-Rad VariantⅡanalyzer (Bio-Rad Laboratories, Hercules, CA) with a reference value of 4.1-6.5%, which is standard in the Diabetes Control and Complications Trial (DCCT) and National Glycohemoglobin Standardization Program (NGSP) [18]. The intra-assay coefficient of variation (CV) was 2.5% and the inter-assay CV was <4.0%, both of which were within the limits of the NGSP [19]. 

### Definition for stroke recurrence

‘Stroke recurrence’ was defined as a deterioration of the previous deficit or a new neurological deficit, which met the definitions for ischemic or hemorrhagic stroke [20]. All recurrence events were identified through rehospitalization diagnosis certification. All abnormalities caused by edema, hemorrhagic transformation, or intercurrent illness were not considered stroke recurrence. Patients who died with recurrent strokes were regarded as cases of stroke recurrence.

### Follow-up

#### Telephone interviews were conducted at 3 months and 1 year

There were 4 trained research personnel who were designated by Jingcheng Company, a clinical research organization. Jingcheng company and the ACROSS study organization executive committee trained the 4 research personnel to ensure the consistency of the interview questions and validity of the interview data. Telephone interviews were performed by the 4 trained research personnel at 3 months and 1 year after the first-ever stroke in the interview centre at Beijing Tiantan Hospital. The interview content was in accordance with the follow-up section in the case report form of the ACROSS-CHINA study. Stroke recurrences associated with rehospitalisation were sourced to the attended hospitals to ensure a reliable diagnosis. For example, if a patient answered a ‘yes’ to experiencing a recurrent event, the name of attended hospital and the attended department (e.g. neurology department) would be sourced. The doctor of the according department would be contacted to check the patient’s information (name, gender, age, identification number, etc.) with the diagnosis certification in the patient list either from the computed system of all the past or present hospital patients or from the registration book of the patients. All the interview data was recorded in the follow-up section of the case report form. During the course of the ACROSS study, clinical research associates, as the third party, designated by the clinical research organization and ACROSS study data monitoring committee supervised the validity of all the data of ACROSS-CHINA study.

### Statistical analysis

Patient baseline characteristics were compared between the stroke recurrence group and recurrence-free group. Age, waist circumference, BMI, blood pressure, FPG, HbA1c, creatitine, high density lipoprotein, homocysteine, and uric acid were expressed as means ± standard deviations; t-test was used for comparison between groups. Gender, tobacco use, alcohol intake, stroke subtypes, past medical history of hypertension or coronary heart disease, oral drugs including antithrombotic agents, anti-hypertension agents and lipid-lowering agents were expressed as frequencies and ratios; χ^2^ test or Fisher’s exact test was used for comparison between groups. All these above variables were included in the univariate analysis. The candidate variables that were entered into the multivariate analysis were based on newly prior published, traditionally or clinically associated with stroke (mainly focused on stroke recurrence), or P<0.10 in the univariate analysis. After a selection of all the variables (detailed in Text S2), the variables entered into the multivariate analysis were: age, gender, education status received, tobacco use, alcohol consumption, systolic and diastolic pressure at baseline and discharge [21], BMI and waist circumference [22], history of coronary heart disease, history of hypertension and history of family stroke [22,23], history of diabetes, ischemic stroke subtypes [24], OCSP subtypes [25], HOMA [26], uric acid [27], homocysteine [28], creatinine [29], high density lipoprotein [29], low density lipoprotein [22], triglyceride and cholesterol [22], FPG, medication therapy (antithrombotic, antihypertensive and lipid-lowering medications) during hospitalization and medication adherence (antithrombotic, antihypertensive and lipid-lowering medications) during follow-up. In the multivariate analysis, gender, tobacco use, alcohol intake, family history of stroke, past medical history of hypertension and coronary heart disease, TOAST and OCSP subtypes, medications’ information, and HbA1c were entered as categorical variables. Age, BMI, waist circumference, blood pressure at baseline and discharge, high density lipoprotein, low density lipoprotein, triglyceride, cholesterol, creatinine, uric acid, HOMA2-IR and homocysteine were entered as continuous variables. FPG was additionally adjusted as a continuous variable to better assess the effect of PSGS on stroke recurrence. Age- and gender- adjusted Cox regression models were first established, and Models 1 and 2 were established for 3-month and 1-year analysis. Model 1 and Model 2 were distinguished depending on whether FPG was enrolled for adjustment. Statistical analyses were performed with the SPSS 17.0 software (SPSS Inc., Chicago, IL). A P <0.05 was considered statistically significant. 

## Results

Of all 2186 patients with first-ever AIS, 1817 patients with non-cardioembolic AIS were available for 3-month follow-up, among which 2 patients died from recurrent stroke and 180 live patients experienced stroke recurrence. For 3-month stroke recurrence, there were 155 ischemic strokes, 21 intracerebral hemorrhages, 4 subarachnoid hemorrhage and 2 other vascular events. For 1-year stroke recurrence, there were 178 ischemic strokes, 31 intracerebral hemorrhages, 6 subarachnoid hemorrhages and 5 other vascular events. The cumulative rate was 8.3% for 3-month stroke recurrence. One thousand five hundred and forty patients with non-cardioembolic AIS were contacted at 1-year follow-up, of which there were 240 patients who experienced stroke recurrence, including 8 patients who died from recurrent stroke. The cumulative rate was 11.0% for 1-year stroke recurrence. 

### Baseline clinical characteristics for the patients involved in the 3-month and 1-year analysis

Table 1 shows the clinical characteristics of patients who were involved in the 3-month and 1-year analysis. The mean age of patients with recurrent stroke was 3 years older than that of recurrence-free patients at 3-month and 1-year follow-up (P=0.003 and P=0.001, respectively). The baseline FPG and HbA1c levels in patients with recurrent stroke were higher than those without recurrent stroke at 3 months and 1 year (P<0.05 for both). Medication adherence in patients with recurrent stroke had no significant difference with those without recurrent stroke (all P>0.05). In the univariate analysis for 3-month stroke recurrence, the P value was <0.10 for the comparisons of HOMA2-IR, homocysteine, uric acid, waist circumference, BMI and history of diabetes between the patients with and without recurrent stroke; in the univariate analysis for 1-year stroke recurrence, the P value was <0.10 for HOMA2-IR and history of diabetes compared between the two groups. 

**Table 1:** Patient Clinical characteristic for 3-month and 1-year analyses.

**Clinical Characteristic**	**3-month**			**1-year**		
	**Recurrence (n=182)**	**Recurrence-free (n=1635)**	**P**	**Recurrence (n=240)**	**Recurrence-free (n=1300)**	**P**
**Age, y**	65±10	62±12	0.003	64±11	61±12	0.001
**Male, n (%)**	108 (59.3)	1021 (62.4)	0.121 ^a^	130 (54.2)	810 (62.3)	0.093^a^
**Body mass index (kg/m^2^)**	25.5±3.9	24.9±3.6	0.054	25.4±4.3	25.0±3.7	0.182
**Waist circumference (centimetre)**	88.2±9.8	86.6±9.7	0.079	87.8±9.9	86.7±9.7	0.324
**Systolic blood pressure (mmHg)**	149±20	147±21	0.385	148±21	147±21	0.826
**Diastolic blood pressure (mmHg)**	85±11	89±12	0.733	86±12	85±12	0.595
**Fasting plasma glucose (m mol/L)**	8.0±3.7	6.4±2.6	<0.001	7.4±3.4	6.4±2.6	<0.001
**HbA1c (%**)	7.5±2.0	6.5±1.8	<0.001	7.2±2.0	6.5±1.7	<0.001
**High density lipoprotein (m mol/L)**	1.17±0.29	1.18±0.34	0.681	1.18±0.29	1.18±0.39	0.978
**HOMA2-IR**	0.52±0.78	0.78±0.85	<0.001	0.60±0.79	0.78±0.85	0.003
**Creatinine (g mol/L)**	74.29±24.68	77.03±30.13	0.238	75.18±23.69	77.13±30.88	0.376
**Homocysteine (μ mol/L)**	16±7	18±11	0.027	17±10	18±11	0.765
**Uric acid (μ mol/L)**	289±102	307±97	0.017	296±101	306±97	0.180
**Tabacco use, n (%)**			0.526 ^a^			0.770 ^a^
**Never**	97 (53.3)	822 (50.3)		115 (47.9)	649 (49.9)	
**Previous**	13 (7.1)	156 (9.5)		20 (8.3)	121 (9.3)	
**Current**	62 (34.1)	548 (33.5)		69 (28.8%)	438 (33.7)	
**Alcohol intake, n (%)**			0.606^b^			0.868 ^b^
**Never**	112 (61.5)	941(57.6)		132 (55.0)	748 (57.5)	
**Moderate**	32 (17.6)	274 (21.1)		37 (15.4)	218 (16.8)	
**Severe**	27 (14.8)	269 (20.7)		36 (15.0)	206 (15.8)	
**TOAST subtype, n (%)**			0.165^a^			0.292 ^a^
**Large vessel infarction**	115 (63.2)	1036 (63.4)		145 (60.4)	802 (61.7)	
**Small vessel infarction**	57 (31.3)	440 (26.9)		62 (25.8)	358 (27.5)	
**Past medical history**						
**No Diabetes, n (%)**	93 (51.1)	1290 (78.9)	<0.001^a^	141 (58.8)	1026 (78.9)	<0.001^a^
**Hypertension, n (%)**	118 (64.8)	966 (59.1)	0.421^b^	138 (57.5)	754 (58.0)	0.531^b^
**Coronary heart disease, n (%)**	25 (13.7)	183 (11.2)	0.358^b^	28 (11.7)	151 (11.6)	0.762^b^
**Medications during hospitalization, n (%)**						
**Anti-thrombotic**	170 (93.4)	1483 (90.7)	0.587 ^a^	201 (83.8)	1172 (90.2)	0.338 ^a^
**Lipid-lowering**	140 (76.9)	1174 (71.8)	0.491 ^a^	163 (67.9)	934 (71.8)	0.835 ^a^
**Anti-hypertensive**	89 (48.9)	708 (43.3)	0.244 ^a^	106 (44.2)	548 (42.2)	0.227 ^a^
**Highadherencewithmedications**						
**3-monthfollow-up^c^, n(%**)						
**Anti-thrombotic**	80 (44.0)	650 (39.8)	0.824 ^a^	N/A	N/A	N/A
**Anti-hypertensive**	50 (27.5)	401 (24.5)	0.155 ^a^	N/A	N/A	N/A
**Lipid-lowering**	30 (16.5)	287 (17.6)	0.254 ^a^	N/A	N/A	N/A
**1-year follow-up ^c^, n(%**)						
**Anti-thrombotic**	N/A	N/A	N/A	71 (29.6)	520 (40.0)	0.345 ^a^
**Anti-hypertensive**	N/A	N/A	N/A	52 (21.7)	298 (22.9)	0.177 ^a^
**Lipid-lowering**	N/A	N/A	N/A	32(13.3)	220(16.9)	0.460 ^a^

a χ^2^ test.

b Fisher’s exact test.

c High adherence was defined as adherence of ≥75%. Medication adherence was calculated as the cumulative duration of taking drugs divided by the length of the follow-up period. If the patient had stroke recurrence, adherence would be calculated as the cumulative duration of taking drugs divided by the length of the follow-up period before the recurrence event.

HOMA2-IR, the correctly solved computer model for homeostasis model assessment of insulin resistance; TOAST, the Trial of ORG 10172 in Acute Stroke Treatment. N/A, not applicable.

### HbA1c results and the 3-month and 1-year stroke recurrence depending on HbA1c quartiles

**Figure 2:**
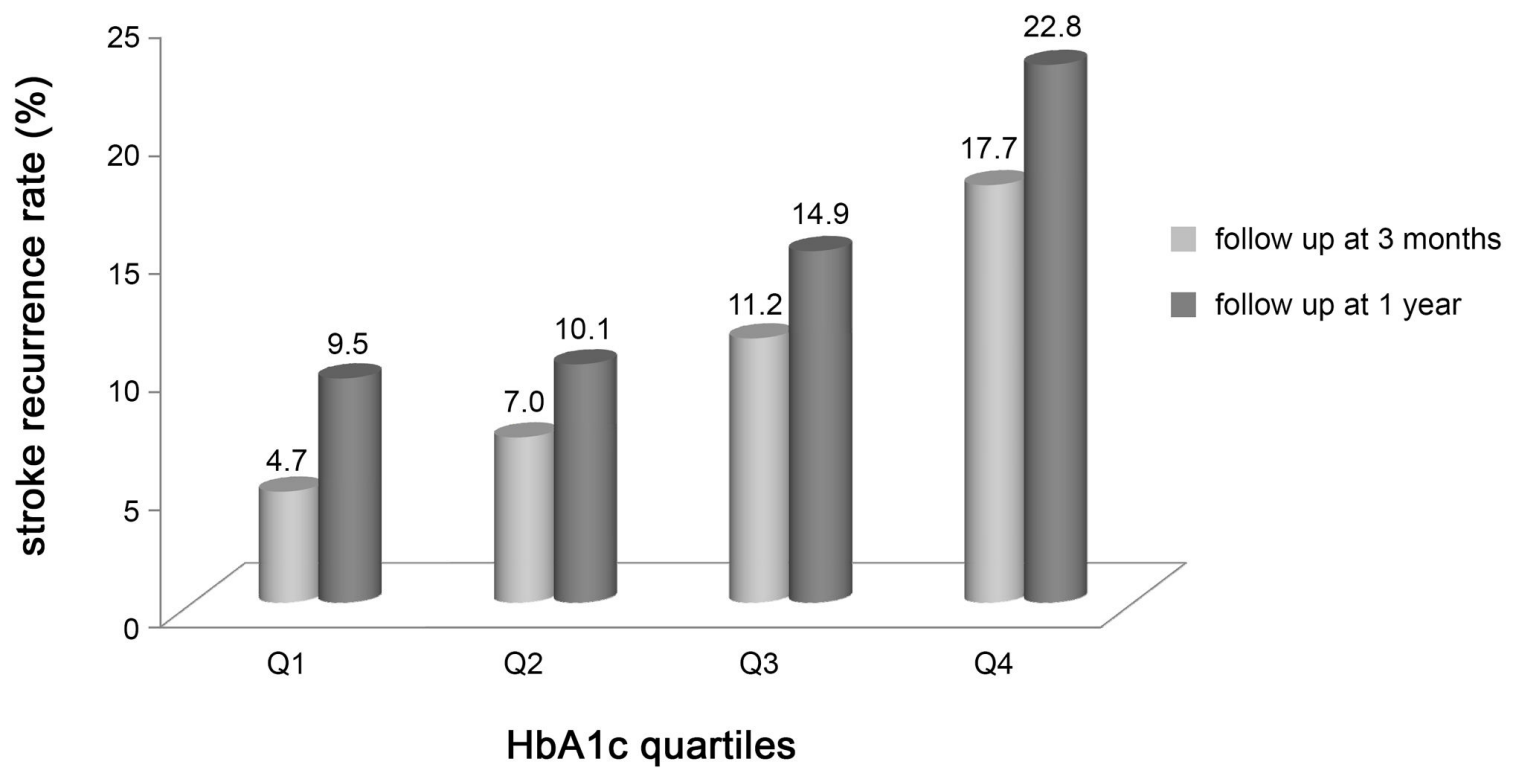
HbA1c levels and number of patients with stroke recurrence. Q1, HbA1c level of <5.5%; Q2, HbA1c level of 5.5 to <6.1%; Q3, HbA1c level of 6.1 to <7.2%; Q4, HbA1c level of ≥7.2%.

The mean level of HbA1c at admission was 6.61% (standard deviation 1.84%) and 6.60% (standard deviation 1.81%) for 1817 patients at 3 months and for 1540 patients at 1 year, respectively. The HbA1c values were divided into 4 levels by quartiles as follows: quartile 1 (Q1): HbA1c <5.5%; quartile 2 (Q2): HbA1c 5.5% to <6.1%; quartile 3 (Q3): HbA1c 6.1 to <7.2%; quartile 4 (Q4): HbA1c ≥7.2%. Figure 2 shows stroke recurrence rates at 3 months (P<0.001) and 1 year (P<0.001) depending on HbA1c quartiles. At 3 months, the cumulative stroke recurrence rate in the Q1 group was 4.7% which gradually increased in the other 3 quartiles (Q2: 7.0%, Q3: 11.2%, and Q4: 17.7%). At 1 year, the cumulative stroke recurrence rate in Q1 was 9.5% which also gradually increased in the other 3 quartiles (Q2: 10.1%, Q3: 14.9%, and Q4: 22.8%). 

### Cox regression analyses for associations between baseline HbA1c levels and stroke recurrence at 3 months and 1 year

**Figure 3:**
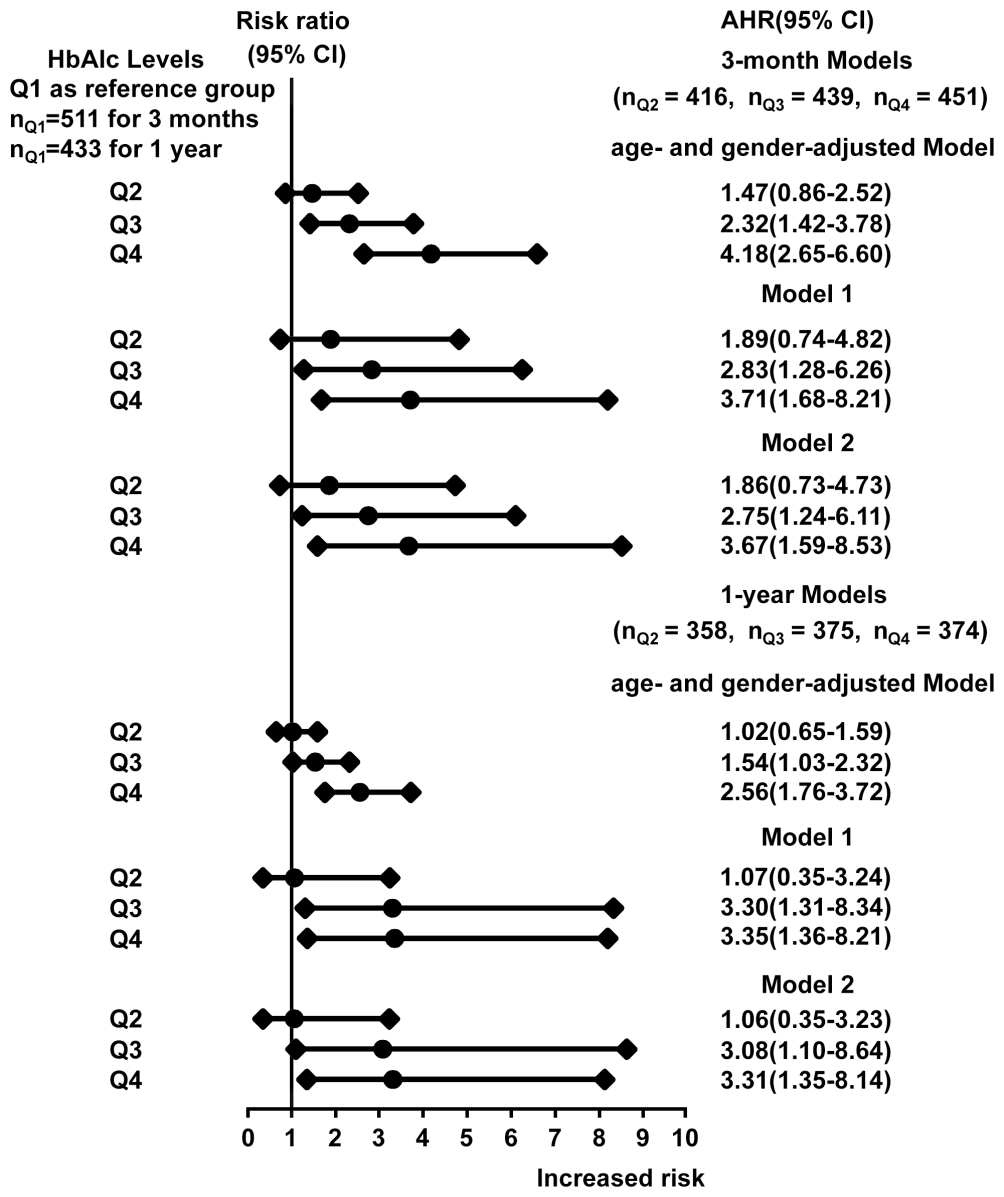
Association between HbA1cl levels and Stroke Recurrence. Q1 (reference group), HbA1c level of <5.5%; Q2, HbA1c level of 5.5 to <6.1%; Q3, HbA1c level of 6.1 to <7.2%; Q4, HbA1c level of ≥7.2%. AHR indicates adjusted hazard ratio; CI indicates confidence interval; HOMA2-IR indicates the correctly solved computer model for homeostasis model assessment of insulin resistance; TOAST, the Trial of ORG 10172 in Acute Stroke Treatment; OCSP, the Oxfordshire Community Stroke Project. 3-month Model 1 adjusted for age, gender, education status received, tabacco use, alcohol consumption, systolic and diastolic pressure at baseline and discharge, BMI and waist circumference, a history of coronary heart disease, a history of hypertension and a history of family stroke, a history of diabetes, ischemic stroke subtypes, OCSP subtypes, HOMA, uric acid, homocysteine, creatinine, high density lipid protein, low density lipoprotein, triglyceride and cholesterol, medication therapy (antithrombotic, antihypertensive and lipid-lowering medications) during hospitalization, and medication adherence (antithrombotic, antihypertensive and lipid-lowering medications) at 3-month follow-up. 3-month Model 2 adjusted for all the variables in 3-month Model 1 plus fasting plasma glucose. 1-year Model 1 adjusted for all the variables in 3-month Model 1 (except 3-month medication adherence) plus medication adherence at 1-year follow up including anti-hypertensive agent, lipid-lowering agent and anti-thrombotic agent. 1-year Model 2 adjusted for all the variables in 1-year Model 1 plus fasting plasma glucose.

In the age- and gender-adjusted Cox regression analyses for both the 3-month and 1-year stroke recurrences, patients with an HbA1c level of ≥6.1% (the lowest limit of Q3) statistically significantly increased the risk of recurrence by 54% to more than 3 times (the minimum AHR was 1.54 and the highest was 4.18) when compared with patients with the HbA1c value of <5.5% (Figure 3). 

In the multiple-adjusted Model 1 (without FPG) for 3 month recurrence, the results were consistent with those in the age and gender-adjusted Cox regression analysis. Patients with an HbA1c level of ≥6.1% had a higher risk than those with an HbA1c level of <5.5% (compared with Q1, AHRs were 2.83 (95% CI 1.28-6.26) in Q3 and 3.71 (95% CI 1.68-8.21) in Q4). In Model 2, FPG was additionally adjusted, and the association between HbA1c and stroke recurrence remained similar (compared with Q1, AHRs were 2.75 (95% CI 1.24-6.11) in Q3 and 3.67 (95% CI 1.59-8.53) in Q4). 

For 1-year multivariate analysis, both Model 1 and Model 2 were also established based on the same rule (Figure 3). Both models showed that the HbA1c level of >6.1% was an independent risk factor for stroke recurrence. In Model 1, when it was compared with patients in Q1, AHRs were 3.30 (95% CI 1.31-8.34) for patients in Q3 and 3.35 (95% CI 1.36-8.21) in Q4. In Model 2, FPG also did not change this association (AHRs were 3.08 (95% CI 1.10-8.64) in Q3 and 3.31(95% CI 1.35-8.14) in Q4). 

After multiple-adjustment, age was associated with the 3-month and 1-year stroke recurrence. Stroke recurrence risk was significantly increased by 3-4% per 1 year at 3 months and 1 year after the initial stroke onset (AHR was 1.03 (95% CI 1.01-1.09) at 3 months; 1.04 (95% CI 1.01-1.07) at 1 year). A history of diabetes was statistically related to stroke recurrence in the multivariate Cox hazard proportional models for 3-month and 1-year analyses (3-month AHR with 95% CI, 2.73 (1.65-4.52) in Model 1, 2.77 (1.65-4.65) in Model 2; 1-year AHR with 95% CI, 2.64 (1.09-6.41) in Model 1, 2.67 (1.10-6.50) in Model 2). FPG was not related to 3-month and 1-year stroke recurrence (3-month AHR with 95% CI, 1.21 (0.95-1.21); 1- year AHR with 95% CI, 1.10 (0.96-1.26)).

### Association between HbA1c at follow-up and stroke recurrence

There were few data on HbA1c at 3-month interval (n=92) and no data on HbA1c available at 1 year. Among the 92 patients, 3 experienced stroke recurrence. The overall glycemic control profile was improved during the 3 months. The mean HbA1c at 3 month was 5.5% (ranged from 4.3% to 13.1%) that was better than that at baseline (6.0% (ranged from 3.8% to 14.6%)) (paired samples T-test, P=0.004). Of all the 92 patients the means of 3-month HbA1c were 5.6% (range from 4.2% to 12.7%) and 6.0% (ranged from 5.5% to 6.4%) for recurrence-free strokes and recurrent strokes respectively (P=0.511), which were ameliorated than those at baseline (5.8% (ranged from 3.8% to 8.8%) for recurrence-free strokes (paired samples T-test, P<0.001) and 9.0% (ranged from 5.6% to 14.6%) for recurrent strokes (paired samples T-test, P=0.801).

Using the same HbA1c scale (<5.5%, 5.5 to <6.1%, 6.1 to <7.2%, ≥7.2%), the number of patients with HbA1c ≥6.1% at 3-month interval was 17 (18.5%), which was 12 less than that at baseline (31.5%) (Table S1). 

In order to investigate whether such a longitude change of HbA1c contributed to 3-month and 1-year stroke recurrence, Cox regression models were built separately to assess the association between baseline/3-month HbA1c and stroke recurrence.

The result showed that 3-month HbA1c, which was entered as a continuous variable, was not significantly associated with either 3-month (HR 1.23, 95% CI 0.66-2.31) or 1-year stroke recurrence (HR 1.28, 95% CI 0.67-2.46), while the baseline HbA1c was statistically significant to 3-month (HR 2.09, 95% CI 1.15-3.82) and 1-year stroke recurrence (HR 1.65, 95% CI 1.18-2.29). However, such an association between 3-month HbA1c and stroke recurrence was most likely contributed to by the small sample size (n=92).

Because of the limited data on 3-month HbA1c, the corresponding information of the longitude change of HbA1c was not included in the multivariate analysis for 3-month and 1-year stroke recurrence.

### The association between history of diabetes as well as anti-diabetic medication with stroke recurrence

A history of diabetes was defined by a self-report by the patient, and/or previous medical record. The diabetic duration for 3-month and 1-year stroke recurrence analysis ranged from 1 month to 30 years. Anti-diabetic medications were defined as oral hypoglycemic agents or insulin therapy for 6 months. Oral hypoglycemic agents included sulphanylureas, metformin, α-glucosidase inhibitor, insulin sensitizers, sulfonylureas and insulin.

Stroke recurrence was also observed in patients without history of diabetes: 51.1% for 3-month and 58.8% for 1-year stroke recurrence (Table 1). 

Table S2 shows the association between a history of diabetes and stroke recurrence at 3-month and 1-year intervals (all P<0.001). Of all the patients for 3-month stroke recurrence analysis in the present study (n=1817), there were 434 patients with a history of diabetes and 1383 patients without a history of diabetes. Among the patients with a history of diabetes, 89 (48.9%) experienced stroke recurrence; among the patients without a history of diabetes, 93 (51.1%) experienced stroke recurrence.

Of all the patients included in 1-year stroke recurrence analysis in the present study (n=1540), there were 373 patients with a history of diabetes and 1167 patients without a history of diabetes. Among the patients with a history of diabetes, 99 (41.3%) experienced stroke recurrence; among the patients without a history of diabetes, 141 (58.8%) experienced stroke recurrence.

In the overall patients without a history of diabetes, further analysis of the association between HbA1c levels and stroke recurrence was performed (Table S3). HbA1c was still associated with stroke recurrence when stratified as ≥6.1% and <6.1%. Compared with patients with HbA1c <6.1%, those with HbA1c ≥6.1% occurred much higher rate of stroke recurrence (P<0.001), 57 (61.3%) recurrent stroke for 3 months and 72 (51.1%) for 1 year respectively.

Among the patients with a history of diabetes, previous oral hypoglycemic agents and insulin administration showed no statistically significant association with 3-month and 1-year stroke recurrence (Table S4). 

## Discussion

In the present study, an HbA1c level of ≥6.1% but not 6.5% independently increased risk for stroke recurrence within 1 year in patients with initial non-cardioembolic AIS. This is somewhat different with the traditional notion about the HbA1c threshold of risk. Usually the HbA1c value would be noticed until it reaches a level of ≥6.5% that is one of the criteria for diagnosing diabetes [9]. Diabetes has been acknowledged as one of the most essential risk predictors for stroke recurrence [5]. However, we found that a high “normal” HbA1c level was also related to stroke recurrence. Our findings indicated that when the HbA1c level achieved 6.1 to ≤7.2%, the risk for stroke recurrence in patients with first-ever non-cardioembolic AIS were significantly increased by 0.54 to 2.30 times higher than that in patients with the HbA1c level of <5.5%; when the HbA1c level reached 7.2% or higher, the risk was increased by 1.56 to 3.18 times higher than those with the HbA1c level of <5.5%.

### Recurrence rate in ischemic strokes

Up to date, reports on short-term ischemic stroke recurrence are inconsistent. In 2009, Allen et al reported that the 1-year cumulative recurrence rate of ischemic stroke was 9.4% [3]. In 2012 Wang et al reported that the 1-year cumulative recurrence rate in ischemic stroke was 17.7% from the China National Stroke Register [2]. However, it was 11.1% in the current study. Several reasons can explain such a discrepancy in the recurrence rate during the same post-stroke period. First, the study populations differed. The Allen et al study and Wang et al study focused on patients with all types of ischemic strokes while we only targeted patients with first-ever non-cardioembolic AIS. Different ischemic stroke subtypes depending on TOAST have great diversities in several aspects such as pathogenesis, recurrence rate, and risk factors for incidence [24] and recurrence [30]. Second, differences in geography and race contribute to the discrepancy. Allen et al included white and black patients while only Chinese patients were recruited into the current study.

### HbA1c and stroke prevention and prognosis

HbA1c is a biochemical index with several advantages including minimal biological variety and nearly not affected by stress response caused by acute diseases [31], and it has been used as an index for monitoring glycemic control status in large clinical trials and clinical practice. 

The paradox about the HbA1c target value for primary stroke prevention or decreasing mortality has not been settled and the perspectives are constantly updated. Several classic clinical trials include the Action to Control Cardiovascular Risk in Diabetes (ACCORD) trial [32], Action in Diabetes and Vascular Disease: Preterax and Diamicron-MR Controlled Evaluation (ADVANCE) study [33], and Veterans Affairs Diabetes Trial (VADT) [34]. In 2008, in the view of Ismail-Beigi and Moghissi, regarding the occurrence of CVD (including stroke) and their prognosis, patients should be treated differently according to the different duration of diabetes and whether they previously had CVD based on all the results from the 3 studies mentioned above [35]. For the patients with newly confirmed diabetes without a history of CVD, glycemic level should be controlled to normal range or close to normal; while for patients who had a long history of diabetes (8-10 years or more) with a confirmed history of CVD, lowering the glycemic level to a normal or nearly normal level could not decrease the risk for CVD or all-cause death. In 2009, the American Diabetes Association, American Heart Association, and American College of Cardiology Foundation claimed that the HbA1c level should be controlled to <7.0% for preventing macrovascular events and future CVD [36]. In 2010, Selvin et al reported that the HbA1c level would be a strong predictor for CVD in the next 10 years [37]. In 2011, a recent report from ADVANCE suggested that the mean HbA1c level of < 7.0% during follow-up would increase risks for macrovascular events and death [38]. In 2012, the newly analysis from ACCORD, ADVANCE, and VADT indicated that the HbA1c level should be controlled to <7.0% but it should account for various factors, such as duration of diabetes, pre-existing macrovascular disease, hypoglycemic unawareness, and significant comorbidities, and be individualized for each patient [39]. 

Consensus has been reached that the HbA1c level at admission in patients with acute stroke can reflect pre-stroke glycaemia [11,22,40-42]. There have been several studies investigating the association between the HbA1c level at admission and stroke. The results varied. In 2011, Oh et al claimed that the elevated HbA1c level at admission to hospital was associated with ischemic stroke incidence merely in male patients without diabetes [42]. In 2011, Kono et al indicated that stroke recurrence had no association with the baseline HbA1c level but was highly related to lifestyle factors within 3 years after acute mild non-cardioembolic stroke onset [22]. The conclusion was inconsistent with that of the present study. Our findings supported that the baseline HbA1c level was independently associated with stroke recurrence both at 3 months and 1 year after the initial stroke onset. The different study populations and follow-up periods may partly account for the different results between studies. 

Although much research aimed to determine the association between the HbA1c level and stroke incidence or prognosis as mentioned above, we have not identified an HbA1c level for secondary stroke prevention from any study. In 2012, the Standards of Medical Care in Diabetes recommended by American Diabetes Association did not mention a threshold of HbA1c level for secondary stroke prevention and only recommended that a HbA1c level of <7.0% could prevent macrovascular complications. For some special patients such as seniors or those experiencing hypoglycemia, an individualized principle should be adapted and a wider glycemic-controlled goal could be accepted [43]. However, our findings strongly suggest that the HbA1c level at admission has an independent association with stroke recurrence from a level of 6.1%, which does not reach the HbA1c cutoff point for diabetes diagnosis (6.5%). Although an HbA1c target value for stroke secondary prevention still cannot be established depending on our findings, we found that in multiple-adjusted models, patients with a higher “normal” HbA1c (≥6.1%) at baseline had an increased risk of stroke recurrence by >2.0 times at 1 year when compared with patients with a HbA1c level of <5.5%. Furthermore, it seems unreasonable to set up a specific cutoff point of HbA1c for preventing recurrent stroke because the latest notion recommended that for glycemic control in stroke secondary prevention, the principle of individuation should be followed [43]. If patients with an elevated HbA1c at baseline (e.g., HbA1c ≥6.1%) had a worse management of physical exercise in the later follow-up intervals, a worse outcome might be observed (higher risk of stroke recurrence). Meanwhile, if patients with a lower HbA1c level (e.g., HbA1c<6.1%) had a better supervision on physical exercise in the later follow-up intervals, a better outcome might be observed (lower risk of stroke recurrence).Thus, we recommend that the identification of HbA1c target in the stroke secondary prevention should be based on the individualization principle. Future studies should add the physical exercise data to better assess the effect of pre-stroke glycemic status on stroke recurrence. However, our findings result in much wider clinic significance for the baseline HbA1c level than previous studies. The baseline HbA1c from a higher “normal” level (≥6.1%) is a significant risk predictor for stroke recurrence and is more useful than merely a glucose monitoring tool for the primary prevention of CVD or diabetes diagnosis and treatment. This means the HbA1c level has significance not only in the area of primary stroke prevention but also in that of secondary stroke prevention. 

### The previous diagnosis and therapy of diabetes and stroke recurrence

Diabetes is a risk factor for stroke and this is also supported in the present study (AHRs were 2.77 (95% CI 1.65-4.65) and 2.67 (95% CI 1.10-6.50) in Model 2 for 3-month and 1-year stroke recurrence analyses, respectively). However, as is seen from the results of the analysis on a history of diabetes and anti-diabetic medication, a number of patients without a history of diabetes had recurrent stroke. There was no association between anti-diabetic medication and stroke recurrence among patients who previously had diabetes. Moreover, of all the patients without a history of diabetes, patients with HbA1c levels of ≥6.1% had much higher recurrent rate than those with HbA1c levels of <6.1% (P<0.05). Although a history of diabetes is vital for stroke recurrence, concerning about the results above and that a HbA1c level of ≥6.1% of the overall enrolled participants was significantly associated with stroke recurrence in the multiple-adjusted Cox regression models (a history of diabetes included as a variable also), PSGS, measured as baseline HbA1c which is a much less variable and more stable parameter, is a meaningful factor for stroke recurrence in patients with and without a history of diabetes.

### PSGS is more vital than FPG in stroke secondary prevention

FPG was added in multiple-adjusted Model 2 and it did not modify the association between the HbA1c level and stroke recurrence. In Model 2, the elevated FPG was related to the increased risk of stroke recurrence but did not reach statistical significance at 3 months and 1 year. This means that the relationship between stroke recurrence and PSGS is closer than FPG. FPG at baseline partly represents post-stroke glycemic level, which has been investigated for a long time due to its great significance in determine stroke prognosis including stroke recurrence [44,45]. However, with regards to stroke recurrence, our research supports that the PSGS is deserved more attention and investigation than post-stroke glycemic level. 

### The longitude change of HbA1c and stroke recurrence

The post-stroke longitude change of HbA1c stands for post-stroke glycemic control status. The analysis of the limited data on patients who had 3-month HbA1c showed the HbA1c level was ameliorated in both recurrence-free strokes (P<0.001) and recurrent strokes (P=0.801). Although the result of hazard analysis on 3-month HbA1c and stroke recurrence was not statistically significant, we cannot conclude that HbA1c at 3-month is not related to 3-month stroke recurrence because the data was limited (n=92). For the same reason, we could not draw the conclusion that PSGS is much more vital than post-glycemic control status in secondary stroke prevention. However, the present study supports that PSGS is an essential factor in stroke recurrence and is more meaningful than FPG at acute stroke phase to stroke recurrence (FPG showed no significant association with stroke recurrence in the multivariate analyses). Further studies should focus on the longitude change of HbA1c and include it into the multivariate analyses on stroke recurrence for better comparing the effect of pre-stroke glycemic status (measured as HbA1c at baseline) and post-glycemic control status (measured as the mean HbA1c during a period of time) on stroke recurrence. 

### Clinic significance of PSGS

The PSGS expressed by a baseline HbA1c level is a significant risk factor for stroke incidence, prognosis, and recurrence. To better evaluate stroke prognosis and recurrence, the baseline HbA1c level should be routinely examined in all patients with AIS without considering whether these patients have abnormal glycometabolism.

### Advantages and limits

The ACROSS-China study is the largest prospective cohort study on abnormal glycometabolism in patients with AIS. Newly identified confounding risk factors for stroke were entered into our multivariate analysis for ascertaining the independent association between HbA1c levels and stroke recurrence. The rates of patients lost to follow up at 3-month and 1-year intervals were 10.9% and 17.0%, respectively, which are lower than those in similar studies [46]. Despite the above advantages, our research has some limits. Selection bias exists because only patients with the baseline HbA1c values were included. The patients lost to follow up at 1 year had a lower mean value of HbA1c level and FPG that would also cause the selection bias (see Table S5). Because of few recurrence cases during such a short follow-up period and the small sample size, the 95% CIs were broad in the present study. The data on HbA1c at follow-up were limited. Due to the limitation of the study design, some confirmed risk factors for CVD could not be collected, such as a personal lifestyle [37]. Additionally, the period of follow-up was relatively short and more information about stroke recurrence could not be observed.

## Conclusions

A higher “normal” HbA1c level of ≥6.1% at admission is an independent predictor for risk of stroke recurrence within 1 year after initial non-cardioembolic AIS onset. When the HbA1c value increases, the risk of stroke recurrence is increased compared with the patients with the HbA1c level of <5.5%. PSGS expressed as baseline HbA1c is a much more significant factor for stroke recurrence than post-stroke glycemic level. The HbA1c level should be recommended as a routine examination in all acute stroke patients for better predicting stroke prognosis. However, our findings cannot deduce that lowering the baseline HbA1c level can benefit decreasing the risk of stroke recurrence.

## Supporting Information

Table S1 **The longitude change of HbA1c in Q1-Q4 overall and in recurrent stroke at baseline and 3-month interval.** Q1, HbA1c level of <5.5%; Q2, HbA1c level of 5.5 to <6.1%; Q3, HbA1c level of 6.1 to <7.2%; Q4, HbA1c level of ≥7.2%.
(DOC)

Table S2 **The association between a history of diabetes and stroke recurrence.**
(DOC)

Table S3 **The association of HbA1c levels and stroke recurrence among patients without a history of diabetes.**
(DOC)

Table S4 **The association between anti-diabetic medicine and stroke recurrence among patients with a history of diabetes.**
(DOC)

Table S5 **Baseline characteristics of patients with non-cardioembolic ischemic stroke who did or did not have 1-year follow-up data.** FPG, fasting plasma glucose; HDL，high density lipid protein; NIHSS, National Institutes of Health Stroke Scale; SBP, systolic blood pressure; DBP, diastolic blood pressure; TG, triglyceride; Cr, creatinine; CHO, cholesterol; BMI, body mass index; HOMA2-IR, the correctly solved computer model for homeostasis model assessment of insulin resistance.
(DOC)

Text S1 **Medication adherence during follow-up.**
(DOC)

Text S2 **Variables selection.**
(DOC)
