# Supplementary material for: Glycated Hemoglobin Independently Predicts Stroke Recurrence within One Year after Acute First-Ever Non-Cardioembolic Strokes Onset in A Chinese Cohort Study
Source: PLoS One. 2013 Nov 13;8(11):e80690. doi: 10.1371/journal.pone.0080690 (PMC3827473; doi:10.1371/journal.pone.0080690)
Supplement: Table S1 — The longitude change of HbA1c in Q1-Q4 overall and in recurrent stroke at baseline and 3-month interval. Q1, HbA1c level of <5.5%; Q2, HbA1c level of 5.5 to <6.1%; Q3, HbA1c level of 6.1 to <7.2%; Q4, HbA1c level of ≥7.2%. (DOC) [file pone.0080690.s001.doc]

Table S1. The longitude change of HbA1c in Q1-Q4 overall and in recurrent stroke at baseline and 3-month interval

| **HbA1c levels** | **Baseline (n=92)** | **Recurrence (n=3)** | **3-month interval (n=92)** | **Recurrence (n=3)** |
| --- | --- | --- | --- | --- |
| **Q1, n (%)** | 33 (35.9) | 0 | 45 (48.9) | 0 |
| **Q2, n (%)** | 30 (32.6) | 1 | 30 (32.6) | 1 |
| **Q3, n (%)** | 21 (22.8) | 1 | 14 (15.2) | 2 |
| **Q4, n (%)** | 8 (8.7) | 1 | 3 (3.3) | 0 |
