# Supplementary material for: Glycated Hemoglobin Independently Predicts Stroke Recurrence within One Year after Acute First-Ever Non-Cardioembolic Strokes Onset in A Chinese Cohort Study
Source: PLoS One. 2013 Nov 13;8(11):e80690. doi: 10.1371/journal.pone.0080690 (PMC3827473; doi:10.1371/journal.pone.0080690)
Supplement: Table S2 — The association between a history of diabetes and stroke recurrence. (DOC) [file pone.0080690.s002.doc]

Table S2. The association between a history of diabetes and stroke recurrence

| **A history of diabetes** | **3-month (n=1817)** | **Recurrence (n=182)** | **No-Recurrence (n=1635)** | **P** | **1-year (n=1540)** | **Recurrence (n=240)** | **No-recurrence (n=1300)** | **P** |
| --- | --- | --- | --- | --- | --- | --- | --- | --- |
| **Yes, n (%)** | 434 (23.9) | 89 (48.9) | 345 (21.1) | <0.001 | 373 (24.2) | 99 (41.3) | 274 (21.1) | <0.001 |
| **No, n (%)** | 1383 (76.1) | 93 (51.1) | 1290 (78.9) |  | 1167 (75.8) | 141 (58.8) | 1026 (78.9) |  |
