# Supplementary material for: Glycated Hemoglobin Independently Predicts Stroke Recurrence within One Year after Acute First-Ever Non-Cardioembolic Strokes Onset in A Chinese Cohort Study
Source: PLoS One. 2013 Nov 13;8(11):e80690. doi: 10.1371/journal.pone.0080690 (PMC3827473; doi:10.1371/journal.pone.0080690)
Supplement: Table S3 — The association of HbA1c levels and stroke recurrence among patients without a history of diabetes. (DOC) [file pone.0080690.s003.doc]

Table S3. The association of HbA1c levels and stroke recurrence among patients without a history of diabetes

| **HbA1c levels** | **3-month (n=1383)** | **Recurrence (n=93)** | **No-recurrence (n=1290)** | **P** | **1-year (n=1167)** | **Recurrence (n=141)** | **No-recurrence (n=1026)** | **P** |
| --- | --- | --- | --- | --- | --- | --- | --- | --- |
| **<6.1%, n (%)** | 858 (62.0) | 36 (38.7) | 822 (63.7) | <0.001 | 727 (62.3) | 69 (48.9) | 658 (64.1) | 0.002 |
| **≥6.1%, n (%)** | 525 (38.0) | 57 (61.3) | 468 (36.3) |  | 440 (37.7) | 72 (51.1) | 368 (35.9) |  |
