# Supplementary material for: Glycated Hemoglobin Independently Predicts Stroke Recurrence within One Year after Acute First-Ever Non-Cardioembolic Strokes Onset in A Chinese Cohort Study
Source: PLoS One. 2013 Nov 13;8(11):e80690. doi: 10.1371/journal.pone.0080690 (PMC3827473; doi:10.1371/journal.pone.0080690)
Supplement: Table S4 — The association between anti-diabetic medicine and stroke recurrence among patients with a history of diabetes. (DOC) [file pone.0080690.s004.doc]

Table S4. The association between anti-diabetic medicine and stroke recurrence among patients with a history of diabetes

| **Antidiabetic agents** |  | **3-month (n=434)** | **Recurrence (n=89)** | **No-recurrence (n=345)** | **P** | **1-year (n=373)** | **Recurrence (n=99)** | **No-recurrence (n=274)** | **P** |
| --- | --- | --- | --- | --- | --- | --- | --- | --- | --- |
| **Oral hypoglycemic agents** | yes | 294 (67.7) | 65 (73.0) | 229 (66.4) | 0.298 | 249 (66.8) | 66 (66.7) | 183 (66.8) | 0.409 |
|  | no | 140 (32.3) | 24 (27.0) | 116 (33.6) |  | 124 (33.2) | 33 (33.3) | 91 (33.2) |  |
| **Insulin** | yes | 92 (21.2) | 20 (22.5) | 72 (20.9) | 0.784 | 75 (20.1) | 17 (17.2) | 58 (21.2) | 0.636 |
|  | no | 342 (78.8) | 69 (77.5) | 273 (79.1) |  | 298 (79.9) | 82 (82.8) | 216 (78.8) |  |
