# Supplementary material for: Glycated Hemoglobin Independently Predicts Stroke Recurrence within One Year after Acute First-Ever Non-Cardioembolic Strokes Onset in A Chinese Cohort Study
Source: PLoS One. 2013 Nov 13;8(11):e80690. doi: 10.1371/journal.pone.0080690 (PMC3827473; doi:10.1371/journal.pone.0080690)
Supplement: Table S5 — Baseline characteristics of patients with non-cardioembolic ischemic stroke who did or did not have 1-year follow-up data. FPG, fasting plasma glucose; HDL，high density lipid protein; NIHSS, National Institutes of Health Stroke Scale; SBP, systolic blood pressure; DBP, diastolic blood pressure; TG, triglyceride; Cr, creatinine; CHO, cholesterol; BMI, body mass index; HOMA2-IR, the correctly solved computer model for homeostasis model assessment of insulin resistance. (DOC) [file pone.0080690.s005.doc]

**Table S5. Baseline characteristics of patients with non-cardioembolic ischemic stroke who did or did not have 1-year follow-up data**

| **Variables** | **Patients with Follow-up Data (n= 1540)** | **Patients Lost to Follow up (n= 538)** | **P value** |
| --- | --- | --- | --- |
| **Age, y** | 62.7±12.3 | 63.6±12.7 | 0.207 |
| **Male, n (%)** | 940 (61.0) | 205 (38.1) | 0.656 |
| **Education status received, above senior school, n (%)** | 533 (34.6) | 84 (15.6) | 0.015 |
| **Alcohol intake (moderate or heavy), n (%)** | 497 (32.3) | 159 (29.6) | 0.821 |
| **Current smoking, n (%)** | 507 (32.9) | 140 (26.0) | 0.412 |
| **Hypertension, n (%)** | 892 (57.9) | 292 (54.3) | 0.857 |
| **Coronary heart disease, n (%)** | 179 (11.6) | 53 (9.9) | 0.354 |
| **HbA1c (%)** | 6.7±1.9 | 6.2±1.8 | <0.001 |
| **FPG (m mol/L)** | 6.71±2.90 | 6.18±2.49 | 0.002 |
| **HDL(m mol/L)** | 1.18±0.38 | 1.19±0.31 | 0.722 |
| **NIHSS** | 5.3±4.6 | 5.9±5.3 | 0.044 |
| **SBP(mmHg)** | 147.6±21.0 | 146.2±22.0 | 0.299 |
| **DBP(mmHg)** | 85.8±12.2 | 85.7±13.4 | 0.877 |
| **TG(m mol/L)** | 1.84±1.25 | 1.72±1.08 | 0.086 |
| **Cr(g mol/L)** | 76.8±29.6 | 77.4±30.8 | 0.743 |
| **Uric acid(μ mol/L)** | 304.0±98.0 | 313.6±94.6 | 0.110 |
| **CHO(m mol/L)** | 4.88±1.15 | 4.70±1.07 | 0.008 |
| **Homocysteine(μ mol/L)** | 17.6±10.7 | 19.1±11.0 | 0.092 |
| **BMI (kg/m2)** | 25.0±3.7 | 24.2±3.3 | 0.001 |
| **Waist circumference (centimetre)** | 86.6±9.2 | 85.8±10.1 | 0.198 |
| **HOMA2-IR** | 0.97±0.15 | 0.98±0.16 | 0.268 |
| **Pneumonia, yes, n (%)** | 112 (7.3) | 23 (4.3) | 0.938 |
| **Urine infection, yes, n (%)** | 52 (3.4) | 13 (2.4) | 0.475 |
| **In-hospital anti-thrombolic agent, n (%)** | 1373 (89.2) | 465 (86.4) | 0.435 |
| **In-hospital anti-hypertensive agent, n (%)** | 654 (42.5) | 212 (39.4) | 0.923 |
| **In-hospital lipid lowering agent, n (%)** | 1097 (71.2) | 233 (43.3) | 0.118 |

FPG, fasting plasma glucose; HDL，high density lipid protein; NIHSS, National Institutes of Health Stroke Scale; SBP, systolic blood pressure; DBP, diastolic blood pressure; TG, triglyceride; Cr, creatinine; CHO, cholesterol; BMI, body mass index; HOMA2-IR, the correctly solved computer model for homeostasis model assessment of insulin resistance.
