# Supplementary material for: Glycated Hemoglobin Independently Predicts Stroke Recurrence within One Year after Acute First-Ever Non-Cardioembolic Strokes Onset in A Chinese Cohort Study
Source: PLoS One. 2013 Nov 13;8(11):e80690. doi: 10.1371/journal.pone.0080690 (PMC3827473; doi:10.1371/journal.pone.0080690)
Supplement: Text S1 — Medication adherence during follow-up. (DOC) [file pone.0080690.s006.doc]

**Text S1 Medication adherence during follow-up**

The data regarding medication adherence during hospitalization were extracted from the case report form. The trained interviewer had a questionnaire of all agents and asked the patients or caregivers through telephone whether they took each of the medications indicated on the list. If an individual received the medication during hospitalization, ‘yes’ was recorded. The medication adherence was assessed through the telephone interview. For patients who experienced stroke recurrence, medication adherence was calculated as the ratio of the cumulative duration of the medication therapy (antithrombotic, antihypertensive, or lipid-lowering therapy) and the duration of overall follow-up before the recurrence event; For patients who did not have stroke recurrence, medication adherence was calculated as the ratio of the cumulative duration of the medication therapy (antithrombotic, antihypertensive, or lipid-lowering therapy) and the according follow-up period. At the 3- and 12-month intervals after initial stroke, patients were asked whether they had taken antithrombotic, antihypertensive, and lipid-lowering therapy since their last follow-up. A response of ‘yes’ at follow up meant the patient insisted on taking the medication since the last visit. An answer of ‘yes’ at the 3-month follow-up was defined as 3 months’ duration of therapy. An answer of ‘yes’ at 12-month follow-up indicated that a patient had been taking medicine from the 3-month follow-up time point to the 12- month follow-up time point. For example, with regards to the antihypertensive medication, for patients with stroke recurrence, if a patient had a recurrent stroke at 3-month follow up, and responded ‘yes’ to antihypertensive drugs used at 3-month follow-up, the adherence level was calculated as 3/3=100%; If a patient had a recurrent stroke at 12-month follow up, an answer of ‘yes’ used at 3-month follow up, an answer of ‘no’ answer at 12-month, the adherence level was calculated as (3+0)/12=25%; If a patient had a recurrent stroke at 12-month follow up, an anwer of ‘no’ with antihypertensive drugs used at 3-month follow up, an answer of ‘yes’ at 12-month, the adherence level was calculated as (0+9)/12=75%. Among patients without stroke recurrence, for 3-month analysis, an answer of ‘yes’ to antihypertensive drugs used, the adherence level was calculated as 3/3=100%, or the adherence level was calculated as 0/3=0; for 1-year analysis, an answer of ‘yes’ used at 3-month follow up, an answer of ‘no’ at 12-month, the adherence level was calculated as (3+0)/12=25%; for 1-year analysis, an answer of ‘no’ used at 3-month follow up, an answer of ‘yes’ answer at 12-month, the adherence level was calculated as (0+9)/12=75% Medication adherence of ≥ 75% was defined as high and < 75% was defined as low. Patients who did not take any medication during follow-up period were defined as untreated. Medication adherence was included as a category variable (including high, low and not treated three levels) in the multivariable analysis for stroke recurrence.
