# Supplementary material for: Glycated Hemoglobin Independently Predicts Stroke Recurrence within One Year after Acute First-Ever Non-Cardioembolic Strokes Onset in A Chinese Cohort Study
Source: PLoS One. 2013 Nov 13;8(11):e80690. doi: 10.1371/journal.pone.0080690 (PMC3827473; doi:10.1371/journal.pone.0080690)
Supplement: Text S2 — Variables selection. (DOC) [file pone.0080690.s007.doc]

**Text S2. Variables selection**

**The variables from the newly prior publications:** ischemic stroke subtypes [1], HOMA [2], uric acid [3], homocysteine [4], creatinine and high density lipid protein [5] (eGFR was treated as one modifiable variable in the reference #5. eGFR was calculated from age, gender, and creatinine. Because age and gender had been entered into our multivariate analysis, creatinine but not eGFR was selected as one variable to avoid over-adjustment), a history of hypertension and a family history of stroke [6,9], systolic and diastolic pressure at discharge [7] (reference #7 pointed out that if blood pressure was elevated, the blood pressure should be lowered as least under 140/90 mmHg after the acute phase of stroke, so we included the blood pressure values at discharge in the analysis. The mean in-hospital duration was 14 days in the present study), OCSP subtypes [8] (reference #8 indicated that brainstem was at high risk for stroke recurrence, so the clinical stroke subtype ‘OCSP’ was selected in the analysis), BMI, waist circumference, low density lipoprotein, triglyceride, and cholesterol [9] (BMI and Waist circumference were used in the reference #9, lipid profile was advanced in the reference #4, so we also included low density lipoprotein, triglyceride, and cholesterol in analysis.)

1. Petty GW, Brown RD Jr, Whisnant JP, Sicks JD, O Fallon WM, et al. (2000) Ischemic stroke subtypes: a population-based study of functional outcome, survival, and recurrence. Stroke 31: 1062- 1068.

2. Gast KB, Tjeerdema N, Stijnen T, Smit JW, Dekkers OM (2012) Insulin resistance and risk of incident cardiovascular events in adults without diabetes: meta-analysis. PLoS One 7: e52036.

3. Chiquete E, Ruiz Sandoval JL, Murillo Bonilla LM, Arauz A, Orozco Valera DR, et al. (2013) Serum uric acid and outcome after acute ischemic stroke: PREMIER study. Cerebrovasc Dis 35: 168- 174.

4. Toole JF, Malinow MR, Chambless LE, Spence JD, Pettigrew LC, et al. (2004) Lowering homocysteine in patients with ischemic stroke to prevent recurrent stroke, myocardial infarction, and death: the Vitamin Intervention for Stroke Prevention (VISP) randomized controlled trial. JAMA 291: 565- 575.

5. Kuwashiro T, Sugimori H, Ago T, Kamouchi M, Kitazono T et al. (2012) Risk factors predisposing to stroke recurrence within one year of non-cardioembolic stroke onset: the Fukuoka Stroke Registry. Cerebrovasc Dis 33: 141- 149.

6. An YC, Chen YX, Wang YX, Zhao XJ, Wang Y, et al. (2011) Risk factors on the recurrence of ischemic stroke and the establishment of a Cox's regression model. Zhonghua Liu Xing Bing Xue Za Zhi 32: 816- 820.

7. Arima H, Chalmers J (2011) PROGRESS: Prevention of Recurrent Stroke. J Clin Hypertens (Greenwich) 13: 693- 702.

8. Ichikawa H, Shimizu Y, Kuriki A, Murakami H, Mukai M, Kawamura M (2012) The brainstem is at high risk for recurrent noncardioembolic cerebral infarction in association with diabetes mellitus: a hospital-based study. Eur Neurol 67: 26- 32.

9. Kono Y, Yamada S, Kamisaka K, Araki A, Fujioka Y, et al. (2011) Recurrence risk after noncardioembolic mild ischemic stroke in a Japanese population. Cerebrovasc Dis 31: 365- 72.

**The risk factors traditionally and clinically related to stroke:** age, gender, education status received, smoking, alcohol consumption, history of diabetes, hypertension, systolic and diastolic blood pressure, coronary heart disease, [atrial](app:ds:atrial) [fibrillation](app:ds:fibrillation) (because cardioembolic ischemic stroke was excluded in the present study, it was not included in the analysis), medication therapy during hospitalization (antithrombotic, antihypertensive and lipid-lowering medications), and medication adherence during follow-up (antithrombotic, antihypertensive, and lipid-lowering medications).

**The variables from the 3-month recurrence univariate analysis with a P<0.10:** age, fasting plasma glucose, HbA1c, a history of diabetes, HOMA2-IR, homocysteine, uric acid, waist circumference, and BMI.

**The variables from the 1-year recurrence univariate analysis with a P<0.10:** age, gender, fasting plasma glucose, HbA1c, HOMA2-IR., a history of diabetes.

**As is seen from the above, the variables eventually entered into the multivariate analysis were:** age, gender, education status received, tobacco use, alcohol consumption, systolic and diastolic pressure at baseline and discharge, BMI and waist circumference, history of coronary heart disease, history of hypertension and history of family stroke, history of diabetes, ischemic stroke subtypes, OCSP subtypes, HOMA, uric acid, homocysteine, creatinine, high density lipoprotein, low density lipoprotein, triglyceride and cholesterol, FPG, medication therapy (antithrombotic, antihypertensive and lipid-lowering medications) during hospitalization and medication adherence (antithrombotic, antihypertensive and lipid-lowering medications) during follow-up.
